# Modeling the Number of Confirmed Cases and Deaths from the COVID-19 Pandemic in the UK and Forecasting from April 15 to May 30, 2020

**DOI:** 10.1017/dmp.2020.312

**Published:** 2020-09-03

**Authors:** Babak Jamshidi, Mansour Rezaei, Mohsen Kakavandi, Shahriar Jamshidi Zargaran

**Affiliations:** Social Development and Health Promotion Research Center, Kermanshah University of Medical Sciences, Kermanshah, Iran; Mechanical Engineering, Poznan University of Technology, Poznan, Poland; Medical Engineering, Tehran University of Medical Sciences, Tehran, Iran

**Keywords:** case-fatality rate, confirmed case, COVID-19, death, forecast, model, relative increment, time series, UK

## Abstract

**Objective:**

The UK is one of the epicenters of coronavirus disease (COVID-19) in the world. As of April 14, there have been 93 873 confirmed patients of COVID-19 in the UK and 12 107 deaths with confirmed infection. On April 14, it was reported that COVID-19 was the cause of more than half of the deaths in London.

**Methods:**

The present paper addresses the modeling and forecasting of the outbreak of COVID-19 in the UK. This modeling must be accomplished through a 2-part time series model to study the number of confirmed cases and deaths. The period we aimed at a forecast was 46 days from April 15 to May 30, 2020. All the computations and simulations were conducted on Matlab R2015b, and the average curves and confidence intervals were calculated based on 100 simulations of the fitted models.

**Results:**

According to the obtained model, we expect that the cumulative number of confirmed cases will reach 282 000 with an 80% confidence interval (242 000 to 316 500) on May 30, from 93 873 on April 14. In addition, it is expected that, over this period, the number of daily new confirmed cases will fall to the interval 1330 to 6450 with the probability of 0.80 by the point estimation around 3100. Regarding death, our model establishes that the real case fatality rate of the pandemic in the UK approaches 11% (80% confidence interval: 8%–15%). Accordingly, we forecast that the total death in the UK will rise to 35 000 (28 000–50 000 with the probability of 80%).

**Conclusions:**

The drawback of this study is the shortage of observations. Also, to conduct a more exact study, it is possible to take the number of the tests into account as an explanatory variable besides time.

Although the ongoing epidemic of coronavirus disease (COVID-19) has a lower fatality rate than Ebola,^1^ Middle Eastern respiratory syndrome (MERS),^2^ and severe acute respiratory syndrome (SARS),^3^ some estimates suggest that the impact of the COVID-19 pandemic may be comparable to the major influenza pandemics of the 20th century.^1^ The pandemic COVID-19 arrived in the United Kingdom (UK) in early 2020. By March 1, the cases had been detected in England, Wales, Northern Ireland, and Scotland. On March 6, for the first time, all the areas of the UK reported simultaneously new daily cases. In late March, the UK joined the other epicenters – the United States, Spain, Italy, France, Germany, China, Iran, and Switzerland, where the spread of the pandemic was significantly more than other territories. The number of confirmed cases increased rapidly in March. As of April 14, 2020, there have been 93 873 confirmed patients of COVID-19 in the UK, and 12 107 deaths with confirmed infection. Up to the same date, London has lost about 4000 people from COVID-19 and had around 20 000 cases infected by severe acute respiratory syndrome coronavirus 2 (SARS-CoV-2), the virus responsible for COVID-19. It means that other than the UK, there are only 14 countries exceeding London in confirmed cases, and just 10 countries have confirmed deaths from the disease more than London.^4-7^ On April 14, the formal statistics reported that COVID-19 was the cause of more than half of the deaths in London. At that time, the UK has been ranked the sixth country for the cumulative number of confirmed cases, the fifth for the number of deaths worldwide, and the fourth for the number of active cases worldwide.^5^ It seems that the UK, in contrast to the other 8 leading countries, has not faced the peak of the new daily confirmed cases. Therefore, it is expected that the rank of the UK in these indices go upper. In addition, a study forecasts that the new coronavirus could infect up to 60% of the population of the UK, in the worst-case scenario.^8^

Overall, considering all the related statistics and events, it is of the utmost importance to have a model to represent and analyze the propagation of the pandemic in this country. The present paper is based on a time series model to represent the spread of the epidemic. This 2-part time series could model the datasets of spreading the following wide range of diseases:

- SARS epidemic of 2003^9^
- MERS epidemic in South Korea, May 20–July 7, 2018^9^
- Ebola outbreak of 2014–2016^9^
- Propagation of HIV/AIDS from 1990 to 2018^9^
- Spreading of the cholera of 2008–2009 in Zimbabwe^9^
- COVID-19 epidemic in China and 4 of its provinces – Beijing, Guangdong, Shanghai, and Hubei in 2020^9^
- COVID-19 epidemic in the United States in 2020^10^
- COVID-19 epidemic in Iran in 2020^11^

As far as we know, applying 2-part time series in the fields other than statistics is unprecedented, and except for Jamshidi et al.’s studies, the time series models used to represent epidemic datasets are restricted to the ARIMA family. For example, refer to several references.^12-17^ We aim to forecast the number of confirmed cases and deaths for a 46-day period, from April 15 to May 30, 2020. It is worth saying that scientists of Imperial College University present weekly forecasts of the reported number of deaths due to COVID-19 in the week ahead and an analysis of case reporting trends for countries with active transmission.^18^ The last forecast of these was published on April 7, 2020, to forecast the number of deaths and pattern of transmissibility for 42 countries from April 8 to 14. The scientists predicted that the UK would lose more than 5000 people, and the pattern of transmissibility in the UK is rapidly growing.

## MODELS, ESTIMATION, SIMULATION, AND PREDICTION

**FIGURE 1:**
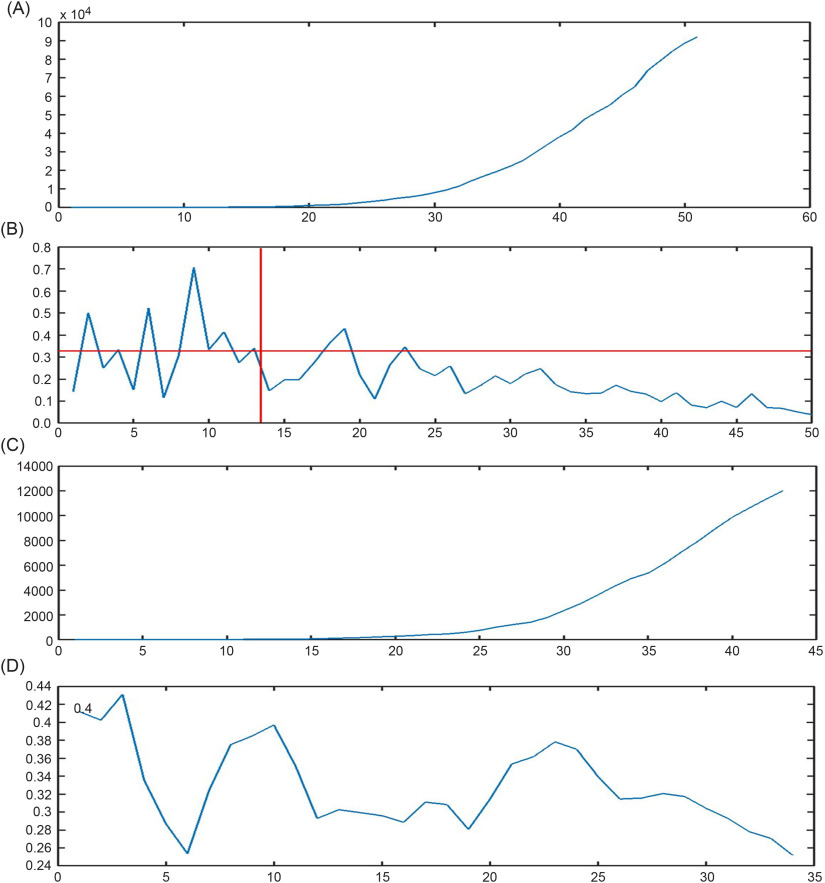
Information about the propagation of COVID-19 in the UK by April 14, 2020. A, Cumulative number of confirmed cases infected by COVID-19 from February 25 to April 14, 2020. B, Time series of RIs and the time of passing from the first stationary period to the new decreasing period (the red horizontal line represents the geometric mean of the RIs that is approximately equal to 0.3272, and the perpendicular line shows the border of the periods (b)). C, Number of deaths from COVID-19 in the UK from 03.03.2020 to 14.04.2020. D, CFR of COVID-19 in the UK from March 11, 2020 to April 14, 2020.

Let 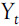 denotes the time series of the number of confirmed cases by the time *t* or cumulative confirmed cases at *t* (Figure 1A). Our objective is to model 
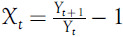
, say the daily relative increment (RI) (Figure 1B). The model that we are going to apply to represent the time series of daily RIs has 5 positive parameters 
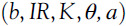
:

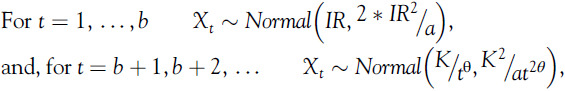

where *b* is the number of days (at the beginning of the spread of the disease) that the daily RI is extreme.^9^ The rationale behind these 2 different patterns is that, after a while, the results of the measurements taken by governments and people to fight against the diseases appear. Figure 1B shows that the time series model is suitable to represent the considered data. *IR* represents the average of the RI in the first period of the spread of the disease when the time series is stationary. After *b* days, the daily RI starts falling regarding the model 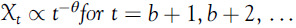. Therefore, 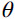 determines the acceleration of the falling of the RIs after the first days of spreading. Also, *a* indicates the fixed ratio of the square of the mean to the variance 
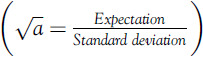
, and, finally, *K* is the adjusting coefficient for the curve 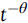 to fit the time series of the RI after the first period of the spread.^9^

In all plots, the x-axis represents the days to April 14, 2020 (daily).

Notably, in the first formula, multiplying by 2 is done due to the higher fluctuation in the first period in comparison with the next days when the time series is smoother. There is another reason for justifying multiplying by 2; in the first days, the behavior of the time series of RI is more chaotic because the numbers are relatively small, so the changes in the ratios are more observable.

To estimate the parameters of the model, we

1. Take *b* as the first point that the geometric mean of the RIs in the previous points exceeds 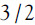 times the geometric mean of the next three points.
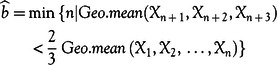
or, 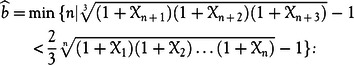

Graphically, this turning time usually can be identified as the time when the plot of RIs falls irreversibly (Figure 1B). According to Figure 1B, 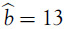, and the perpendicular red line depicts this turning time.
2. Calculate the geometric mean of the RIs from 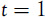 to 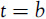 as the estimation of the parameter *IR*:
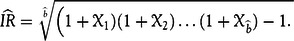

  The horizontal red line of Figure 1B represents the estimation of the parameter *IR*. To some extent, the shift from stage 1 to stage 2 can be seen as a shock because it affects both the mean and the variance.
3. Obtain the estimation of the parameters 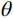 and *K* by using the following linear relation:
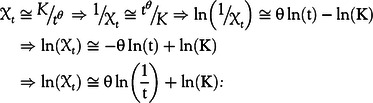

  Due to the classification of the periods during the calculation of the estimation of *b*, it is concluded that 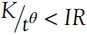 for all 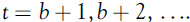.
Multiply all the observations after 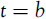 by 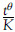 to have an identical mean and variance for all of the newly obtained data (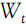):
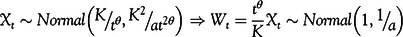

  Therefore, the variance of the newly obtained data is a good candidate for estimating 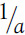. So 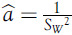 [9].

Accordingly, we get the following estimations to simulate the time series of daily RIs from February 25 to April 14, 2020, by the model:
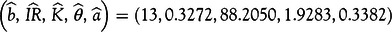



Now, we are going to model the ratio of the deaths (Figure 1C) to confirmed cases (Figure 1A), which is called the case fatality rate or case fatality ratio (CFR):
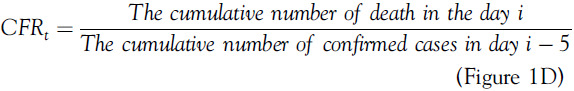



Modeling the time series of CFRs is conducted by the adjusted form of the second part of the above model. As the CFR follows a falling trend, and the data
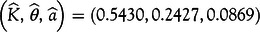
regarding COVID-19 worldwide has shown a short-term stabilization point around 7% for the mean of CFR, we use the average of short-term average and the decreasing model:
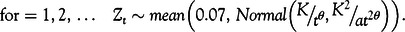



Calculating the distribution, mean, and variance of 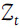, it is concluded that
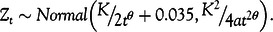



Therefore, we apply a similar method to find the estimate of the parameters. Accordingly, we obtain the following estimates for the model to represent the CFRs from March 13 to April 14.

All the simulations and calculations in the present work have been done by means of Matlab R2015b, and the average curves and confidence intervals are calculated based on 1000 simulations of the fitted models.

According to Figure 1B, the time series of RIs had experienced its extreme values during 13 days after February 25, while it had been fluctuating around the line introducing the geometric mean of the RIs. Thereafter, the time series had a slight downward trend and almost experienced fewer number than 0.33 in the second period: from the 13th day until April 14.

**FIGURE 2:**
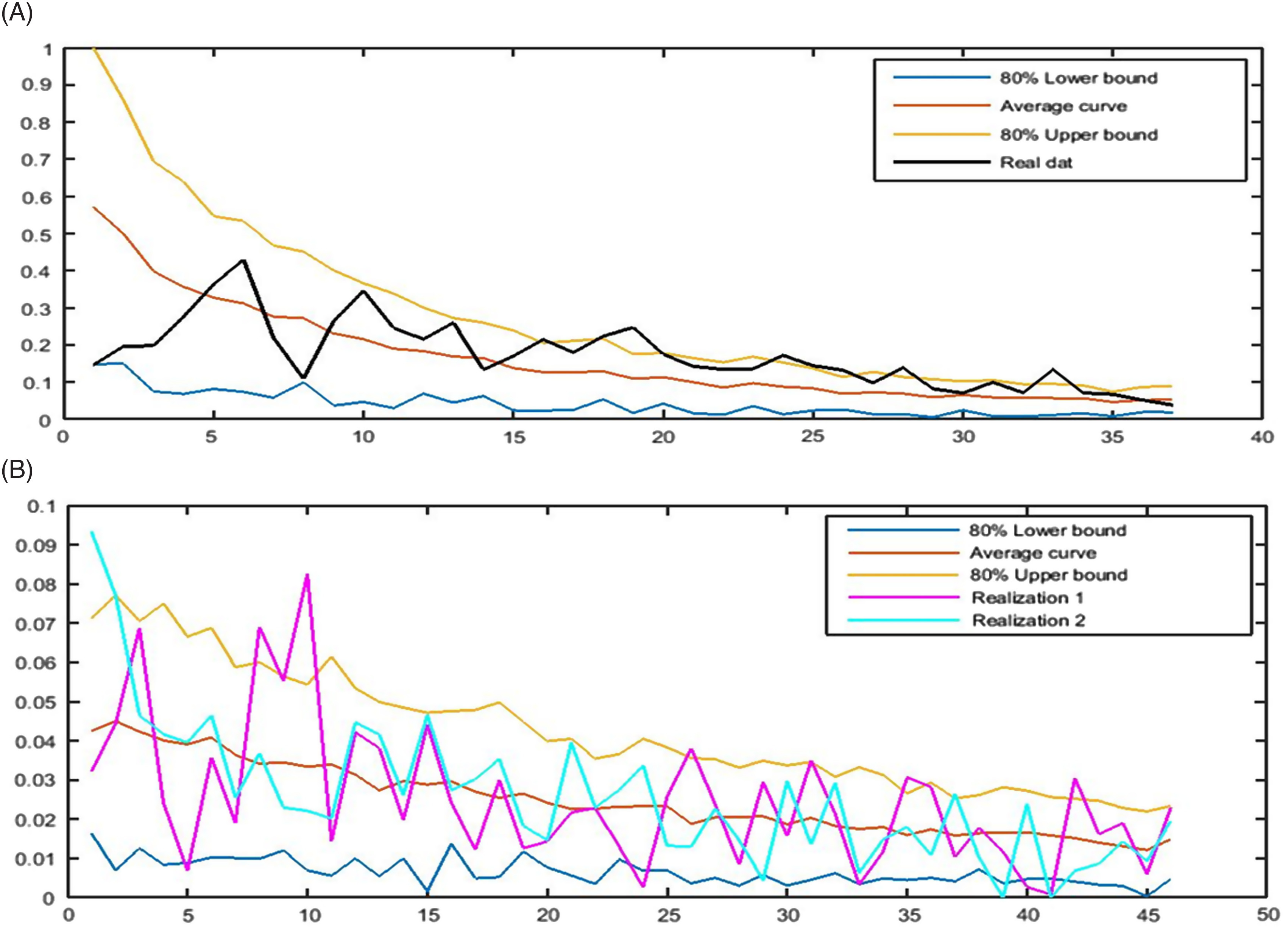
Fitting RIs by the model. A, The real RIs of the number of confirmed cases, the 80% confidence interval, and the average curve, 09.03.2020–14.04.2020. B, The predicted 80% confidence interval, the average curve, and 2 realizations of the RIs based on the model from April 15, 2020 to May 30, 2020.

Figure 2A illustrates how the obtained model fit the real daily RIs of confirmed cases infected by COVID-19 from March 9 to April 14. It is observable that the trend of the real data is similar to the pattern of the bounds and average curves. The model predicts that RI is decreasing from around 4.5% to about 1.5%, and its 80% confidence interval on 30.05.2020 is 0.48%–2.34% (Figure 2B).

**FIGURE 3:**
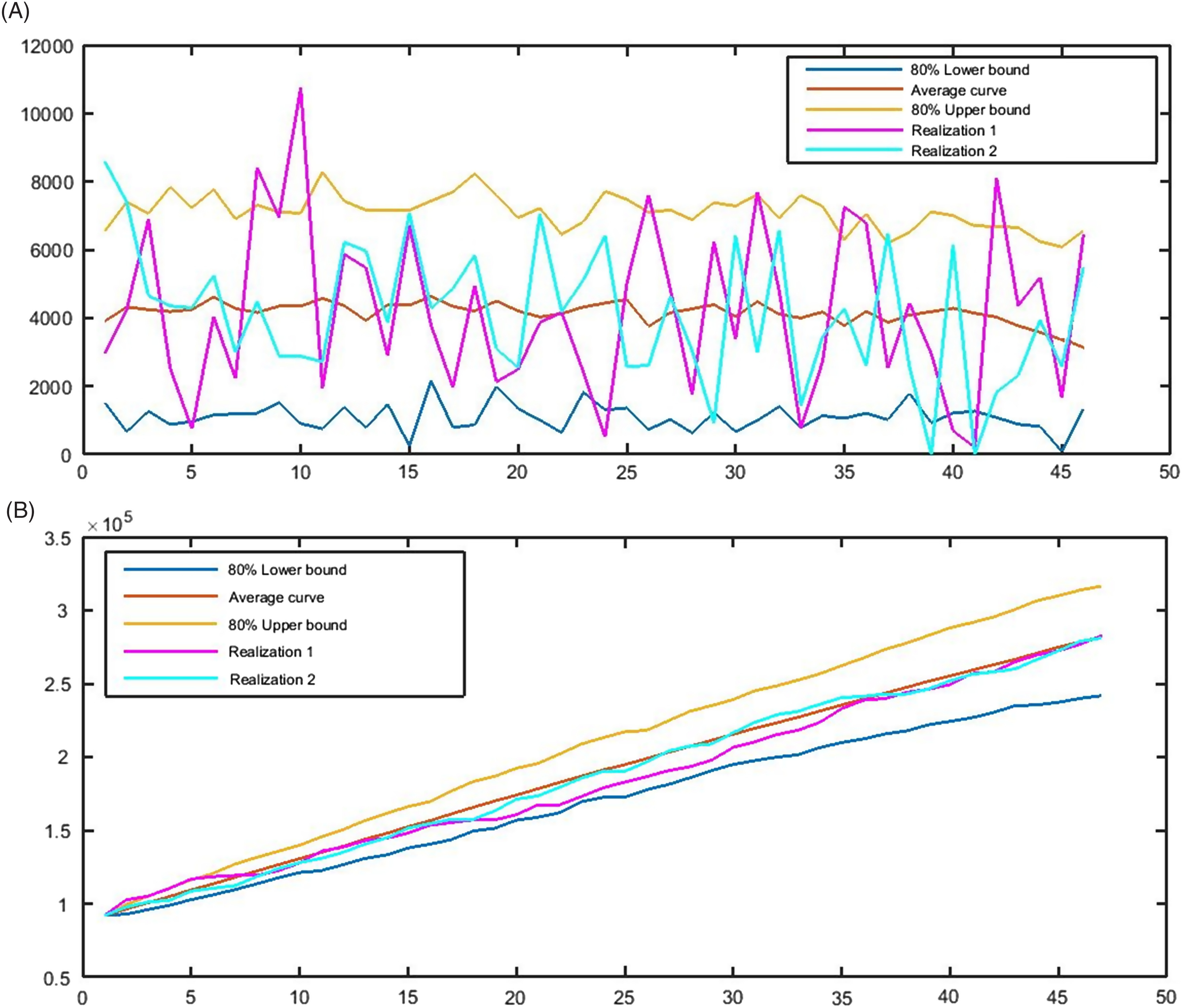
The 80% confidence interval, the average curve, and 2 realizations of the number of A, new daily and B, total confirmed cases based on the model from April 15, 2020 to May 30, 2020.

Despite the increasing scheme of the cumulative number of confirmed cases, the trend of daily RI is downward, and the count of new confirmed cases follows a falling pattern. In the first half of April, the count had been fluctuating around 4500, while based on the model, it is predicted that the number of new cases decreases to around 3100 as the point estimation and 1330–6450 with the probability of 80% on May 31, 2020 (Figure 3A). Based on the obtained results, the cumulative number of cases infected by COVID-19 starts rising from 93 873 on April 14 to 282 000 cases on May 30, 2020, with 80% confidence interval equal to 242 000–316 000 (Figure 3B).

**FIGURE 4:**
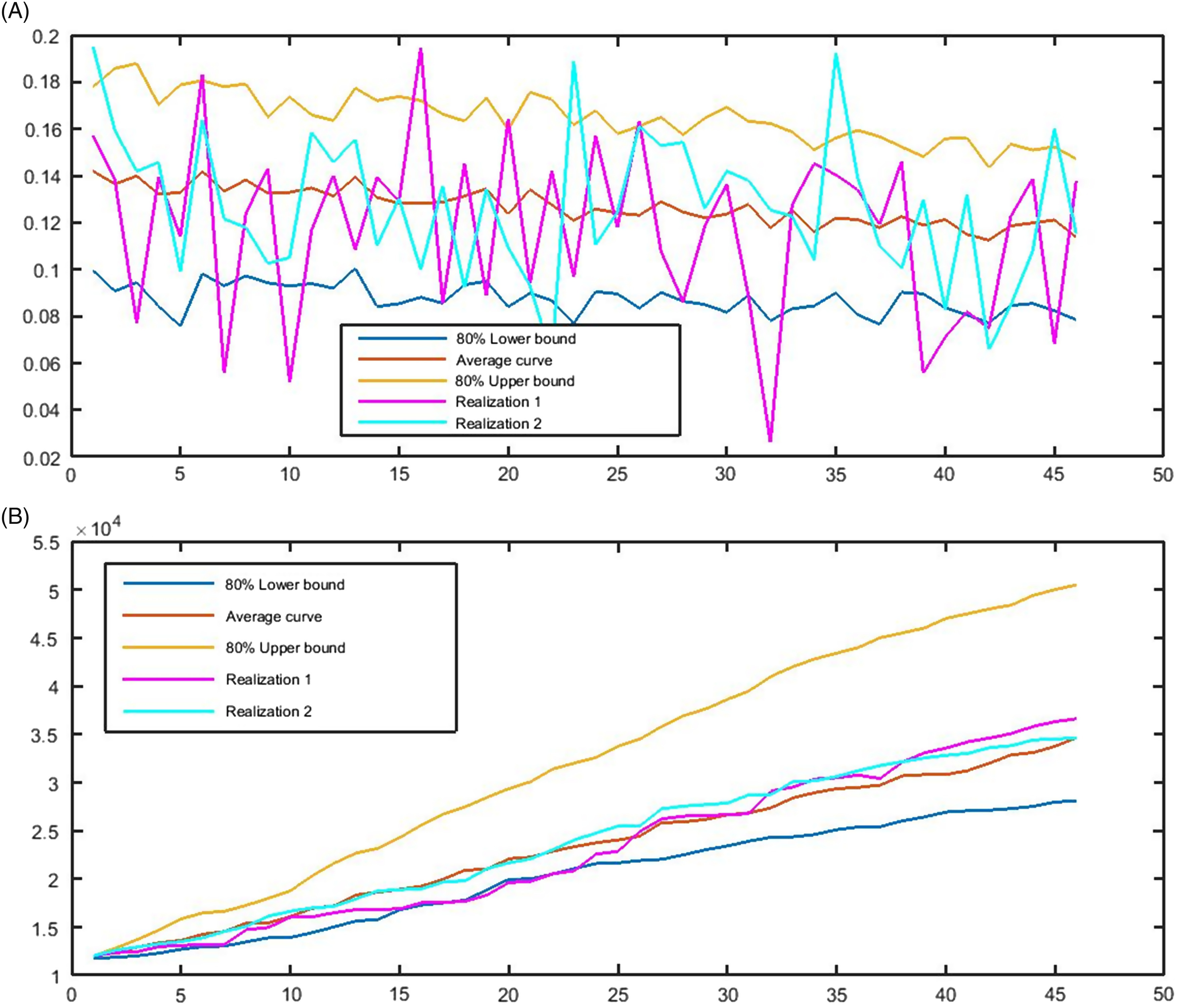
A, The 80% confidence interval, the average curve, and 2 realizations of the CFR; B, cumulative number of deaths based on the model from April 15, 2020, to May 30, 2020.

According to the model representing the data concerning the CFR, the trend of the CFR in the next 46 days will be decreasing by a mild acceleration. Our forecast establishes that the CFR is falling around 11% by May 30. The 80% confidence interval for this index was obtained at 8–15% (Figure 4A). Therefore, we expect that almost 11% of 282 000 confirmed cases will die from the pandemic in the UK by the end of May 2020. This means that the UK will see around 31 000 deaths up to May 30. Figure 4B promotes this raw forecast. According to Figure 4B, the UK will lose around 23 000 people dying from COVID-19 in May and the second half of April 2020. Accordingly, the interval of 28 000 to 50 000 deaths is obtained for the total death up to the end of May 2020.

## CONCLUSION

On March 16, 2020, the UK Government announced new actions to control COVID-19. These actions include self-isolation for 14 days, social distancing, working at home, and stopping unnecessary travel as far as possible, accompanied by the suspension of mass gatherings, such as sporting events and some activities like allocation budget for research on the pandemic and international scientific collaboration with other countries like China.^19^ Surveys have shown that most people have done the government recommendations on COVID-19, and considering this behavior is well reflected by the shift from stage 1 to stage 2 in Figure 1B. However, the acceleration of the spread of this disease is high, and this high rate of transmission caused the activities in the UK to become suspended. Since it is of the utmost importance to have a model to forecast the spread of the disease, especially for health policy-makers and governors, the present paper provided the prediction of the number of deaths and confirmed cases by COVID-19 from April 15 to May 30, 2020.

Notably, our analyses were based on the assumption that the actions taken by the government and people do not considerably differ from the previous interventions, and we will not face an extremely severe lockdown or a society with quite ordinary relationships (such as before the ongoing pandemic). Strictly speaking, the basis of the model is the average scenario (neither extremely fast nor extremely slow) of the growth. According to our results, it is expected that the cumulative number of confirmed cases will have risen to 282 000 (with a 80% confidence interval of 242 000 to 316 500 by May 30, and the number of daily new confirmed cases falls to the interval of 1330 to 6450 with the probability of 0.80 (with the point estimation around 3100). Also, our model establishes that the CFR of the pandemic in the UK approaches 11% (80% confidence interval: 8%–15%) over the coming 46 days. Accordingly, the total death in the UK rises to 35 000 (28 000–50 000 with the probability of 80%). Logically, taking much more or much less severe interventions leads to the exit of the studied indicators from the predicted bands from downward or upward, respectively.

The drawback of this study is the shortage of observations because, in a time series, the observations must be significantly more than the forecast period. Also, in order to conduct a more general and more exact study, it is possible to take the number of the tests into account as an explanatory variable besides time. In addition, it is suggested to model the number of deaths and cases based on just the number of tests as the only explanatory variable (regression analysis) or conduct a time series modeling to analyze the number of tests. Notably, these ideas are applicable to the countries where all tests are recorded in time. Finally, addressing the existence of shocks among the studied dataset (eg, COVID-19 in China) and how to justify the model to fit them can be a research of interest for future statistical studies.
